# Deploying Elemental Iodine in a Vapor Form to Disinfect Water and to Clear Biofilms

**DOI:** 10.3390/ijerph17103489

**Published:** 2020-05-16

**Authors:** Petronella R. Hove, Daniel Mobley, Forgivemore Magunda, Douglas R. Call

**Affiliations:** 1 Department of Microbiology, Immunology & Pathology, Colorado State University, Fort Collins, CO 80523, USA; forgivemore.magunda@colostate.edu; 2 Paul G. Allen School for Global Animal Health, Washington State University, Pullman, WA 99163, USA; dan.mobley@email.wsu.edu (D.M.); drcall@wsu.edu (D.R.C.)

**Keywords:** iodine, sanitation, elemental iodine, iodine vapor infusion, water treatment, wastewater treatment

## Abstract

Traditionally, iodine has been delivered as a solution, tablet or resin to disinfect water. In this study we evaluated the “I_2_ vapor infusion” (I_2_VP) technology which passes an airstream through a matrix containing elemental iodine (I_2_) to produce I_2_ vapor as an innovative method of iodine delivery for water disinfection. Pressured air was provided either by a compressor or hand pump. Testing was performed with water inoculated with either Gram-negative (*Escherichia*, *Salmonella*) or Gram-positive (*Enterococcus*) bacteria or with pre-formed *Acinetobacter* or *Staphylococcus* biofilms. Bacterial colony forming units were used to assess efficacy of the device. In distilled water all bacteria and biofilms were eliminated after brief exposures (<90 s). Culturable bacteria were also eliminated from pond and municipal sewer water, but the technology was mostly ineffective against dairy lagoon water with high turbidity and organic particulate. Longer duration infusion and higher air volumes used to overcome interference from organic matter were also associated with higher concentrations of residual iodine. We conclude that I_2_ vapor infusion has the potential to be useful for emergency water treatment and potentially for reducing microbiological contamination of some waste streams.

## 1. Introduction

Unclean drinking water is an important source of pathogenic bacteria, viruses and protozoan parasites for 2.7 billion people worldwide, underscoring the need for innovative safe and effective water disinfecting methods [1]. Current methods of water disinfection broadly include physical or chemical treatment. Among the well-known chemical disinfectants are halogens, which include bromine, fluorine, chlorine and iodine. [2,3]. Of these iodine has been widely used because it is generally effective, simple, cost-efficient and its properties for water disinfection are well known [4].

Iodine has mainly been used in emergencies and by travelers [4,5]. Its use as a water disinfectant can be dated back to the early 1900 s when the military first developed a tablet formulation for use in the field [6]. Like all halogens, iodine is a biocide because it is a strong oxidant [4]. However, relative to other halogens, complexed iodine has greater chemical stability [7]. It is also less reactive with organic nitrogenous contaminants and can be retained at a higher residual concentration in water [7]. These characteristics mean that iodine residuals will persist longer and will be more stable in the presence of organic matter compared to chlorine, the other commonly used halogen for water disinfection [6,8]. Compared to chlorine, iodine also has a more acceptable taste in equipotent concentrations and it is effective over a wider pH range [9,10,11]. Although the exact mechanism of action is unknown, iodine is known to rapidly penetrate into microorganisms [12]. It targets aromatic C–H functions, sulfur-containing amino acids (cysteine, methionine), and unsaturated fatty acids [13]. As a bactericidal agent, iodine likely penetrates bacterial cell walls, and it’s killing mechanism is likely related to retardation of bacterial protein synthesis, disruption of electron transport, DNA denaturation or membrane destabilization [3]. In lipid enveloped viruses [14] it is presumed to attack the surface proteins and destabilize membrane fatty acids by reacting with unsaturated carbon bonds [15]. Povidone iodine has also been reported as an effective environmental microbicide in the inactivation of coronaviruses [16].

In water, iodine is minimally soluble (0.03 g/100 g water at 20 °C) compared to other halogens [17]. When added to water, it may remain unchanged or it may hydrolyze into different species depending on temperature, pH and the initial concentration. These different species include hypoiodous acid (HIO), iodide ion (I^−^), hypoiodite ion (IO^−^), triiodide ion (I_3_^−^), iodic acid (HIO_3_) and iodate (IO_3_^−^) [18]. It also exists as the diatomic elemental molecule, I_2,_ momentarily after being oxidized from iodide. Of the species formed, only I_2_ and HIO are capable of biocide activity [8,9,17,19,20]. A pH near neutral to mildly alkaline (pH 7–7.5) allows for adequate levels of both elemental iodine and hypoiodous acid [7]. The other iodine species serve as “reservoirs” by providing a source for I_2_ [17].

For practical purposes, iodine is employed as an iodine solution (e.g., tincture of iodine, a 2% iodine solution) and as tablets containing iodine along with carrier and stabilizing agents to enhance dissolvability (e.g., Globaline, composed of tetraglycine hydroperiodide, sodium acid pyrophosphate and talc; [20]). Iodine resins have also been designed as solid-phase iodine disinfectants were water disinfection occurs through direct contact with the microorganism as water passes through iodine that is adsorbed onto the resin [4]. The same principle is used in the formulation of iodinated biocides where the degree of disinfection is determined by the availability and regulated release of free elemental iodine [21].

Based on these characteristics of iodine, we propose that presentation of elemental iodine in an un-complexed form coupled to a delivery method that maximizes distribution and interaction with the target microbes would offer significant and rapid antimicrobial activity in a fluid. We evaluated the effectiveness of an “I_2_ vapor infusion” (I_2_VP) technology that introduces free iodine into solutions via transitory air bubbles that contain I_2_ vapor [22] The I_2_VP technology is an adaptation of the iodine-infused aeration of elemental I_2_ for hull fouling prevention. This method uses an infusion of air bubbles containing I_2_ to prevent or reduce fouling by inactivating bacteria that create fouling matrixes [23]. The goal was to determine if I_2_VP was capable of general water disinfection by testing the killing of gram-positive and negative bacteria either in free solution or as biofilm communities. This was considered in water of varying quality. We used the technology in two forms; as a hand powered system for use in emergencies, and for applications relevant to both water and wastewater treatment.

## 2. Materials and Methods

### 2.1. “I_2_ Vapor Perfusion” (I_2_VP) System

**Figure 1:**
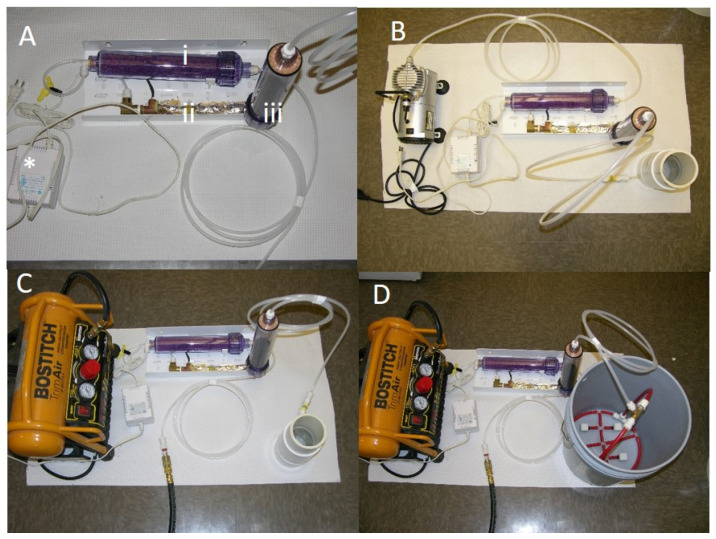
I_2_ vapor infusion (I_2_VP) system with different electric powered compressors. (**A**) Basic components of the I_2_VP system. Order taken by air (**i**) cartridge with moisture trapping beads (**ii**) heated tube to warm up air (**iii**) cartridge containing iodine coated resins. (**B**–**D**) various configurations of the I_2_VP diffuser System. (**B**) Air supplied by a lower capacity pump, Gilford Vacuum Pump (115V 60Hz 2.3A, 58 max psi) attached to a 250 mL cylinder. (**C**) Air supplied by the higher capacity Bostitch trim Air 2.8CFM High Output compressor attached to a 250 mL cylinder. (**D**) Air supplied by the Bostitch trim Air 2.8CFM High Output compressor attached to a 5-gallon bucket. * 48-volt transformer.

The I_2_ fluid innovation system used in this study is a derivation of the device created by I_2_ Air Fluid Innovation as a method for airstream, fluid and vessel decontamination, (Radicone and Miller 2008) [22]. Air was delivered to the I2VP system by either an electric compressor or hand pump. The airstream was first directed to a heater element (Figure 1A, ii.) warmed to 90 °F to increase sublimation of iodine. The amount of air entering the unit was controlled by a manual valve that was fixed to one position for all the experiments. From the heater element, air then entered the iodine resin encased in a cartridge. (Figure 1A, iii.). The iodine cartridge comprises of an air-inlet portion, through which air enters. The column of the cartridge is packed with beaded iodine resin. As air passes from the air-inlet portion, into the column, the iodine resin rapidly sublimates iodine ions into the air passing through the column and exists at the distal end connected to a tube. Iodine vapor is then delivered by the tube into a bubble forming element at the bottom of the container (Figure 1B–D) with water to be disinfected. The bubble forming element is constructed from a porous material and provides active bubble formation. The iodine-laden air bubbles sublimate the iodine into the fluid treating microbial contamination that may be present in the fluid [22].

For the compressor system (Figure 1) air was supplied through a low-capacity compressor, Gilford Vacuum Pump (115V 60Hz 2.3A, 58 max psi) attached to a 250 mL cylinder (Figure 1B).

For the experiments described this compressor was operated at maximum (58 psi). A high-capacity Bostitch trim Air 2.8CFM High Output compressor attached to a 250 mL cylinder or 5-gallon bucket. This compressor was operated between (75–100 psi).

**Figure 2:**
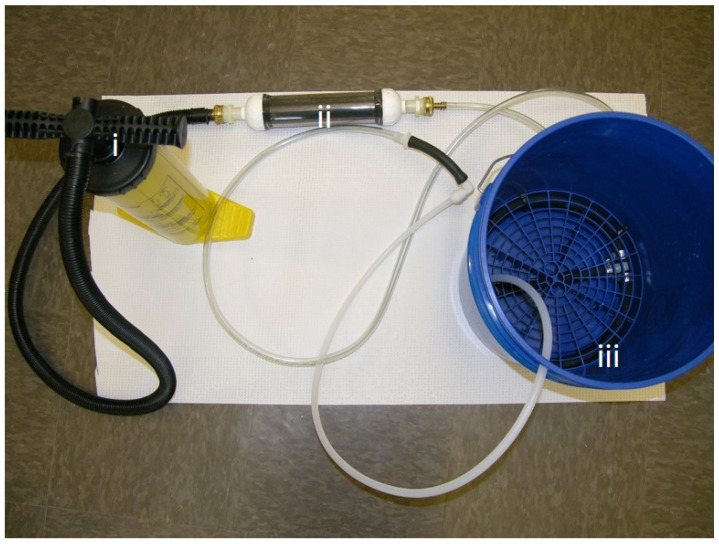
Configuration of the I_2_VP manually powered ‘bucket system’. A hand pump (**i**) attached to the cartridge (**ii**) containing iodine coated resin. When air is pumped through the resin, vaporized elemental iodine is forced through a tube that connects to a coil diffuser system (**iii**) at the bottom of a 5-gallon bucket.

The hand powered (bucket) system (Figure 2). Following initial testing with the electric system we adapted the iodine cartridge to a simple, hand-powered “bucket system” to determine if the technology could be scaled for use in households that lack access to potable water and electricity. The same iodine cartridge design was used for both the electric and hand powered system. Through the action of the hand pump ambient air was passed through the cartridge and delivered into a 5-gallon bucket through a coiled tube diffuser. Air was not pre-warmed with the bucket system. For the bucket system, the total volume of water used was approximately 3 L.

### 2.2. Experimental Set Up

**Figure 3:**
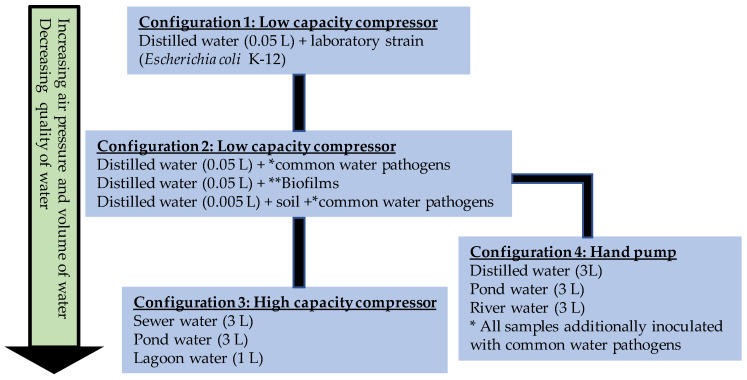
List of experimental setups for the disinfection of bacteria contaminated water using vaporized iodine. Configurations 1 is a proof of concept using water inoculated with a laboratory strain. Configurations 2 to 4 were setup to determine disinfecting efficacy of compressors delivering increasing air pressure and volume of water in disinfecting water of decreasing quality. * Bacterial pathogens tested are *Escherichia coli* O157:H7, *Enterococcus faecalis, Salmonella enterica* ** Biofilms composed of *Acinetobacter baumannii, Staphylococcus aureus.*

Three configurations were used with the two different compressors (Figure 1), and a fourth configuration deployed a hand-operated recreational inflation pump (Figure 2). Experiments were done in increasing level of air pressure and decreasing water quality as determined by visual inspection. The first configuration used the low capacity compressor Gilford vacuum pump (115V 60Hz 2.3A, 58 psi) attached to a 250 mL cylinder with distilled water (i) inoculated with common water pathogens and biofilms and (ii) distilled water plus loess soil to simulate water of low quality containing organic matter (Figure 1B). The total carbon (TC) content was determined with a Leco CNS analyzer (Leco, St. Joseph, MI, USA) described previously [24]. No carbonates were detected, and soil was measured to have a total organic content of 2.27%. The second configuration used the high capacity compressor Bostitch trim Air 2.8CFM High Output compressor (75–100 psi) attached to a 250 mL cylinder (Figure 1C) and centrifuged water (with particulate matter removed) from a diary lagoon. The third configuration was composed of the high capacity compressor attached to a 5-gallon bucket and was used to treat water from naturally occurring water bodies (pond, sewer and lagoon) to simulate use of the technology in real settings with high particulate matter or organic matter. The fourth configuration used a hand pump to treat (i) distilled water inoculated with common water pathogens (ii) water from naturally occurring water bodies to simulate use in low resource settings (water was inoculated with pathogenic bacteria to reach high bacterial load). Summary of the experimental set up is shown in Figure 3.

### 2.3. Bacterial Strains, Inoculation, Conditions and Enumeration

Bacteria causing diseases transmitted through water were tested, these included two *Escherichia coli* strains, *E. coli* O157:H7 ((RIMD 0509952) (Sakai strain; [25,26]) a concern in outbreaks involving consumption of drinking water contaminated with human sewage or cattle feces. A lab adapted strain, nalidixic-resistant *E. coli* K12 (E. *coli* K12 Nal^R^, [27]) was included as a control. *Salmonella enterica* serovar Typhimurium [28,29] an environmental contaminate, commonly found in municipal sewage, agriculture pollution, and storm water; *Enterococcus faecalis* an important opportunistic pathogen frequently found in water [30]. Two biofilm producing strains, multi-drug resistant *Acinetobacter baumannii* (ATCC BAA-1605) and methicillin-resistant *Staphylococcus aureus* (ATCC BAA-1747) were also included. Strains were tested independently in each experimental run.

Bacteria were cultured overnight, and the resulting optical density was measured at 600 nm (OD_600_) to estimate colony forming units (cfu) mL^−1^ and then inoculated into water at a final concentration of approximately 10^6^ cfu mL^−1^ (to ensure a high concentration of bacteria). Before inoculation, cultures were centrifuged at 3000x *g* for 15 min and washed two times by re-suspending in 1X Dulbecco’s Phosphate Buffered saline (PBS; MP Biomedicals LLC, Solon, OH, USA). Inoculum was added directly to water. Before treatment 2 mL samples were collected to determine starting cfu mL^−1^. Water was then treated with iodine using the I_2_VP system.

After treatment, 2 mL water samples were collected to determine colony forming units (cfu) mL^−1^. Serial dilutions were prepared and cfu mL^−1^ was estimated by using a 6X6 drop-plate method [31]. Lennox Broth (LB) (Becton, Dickinson and Company, Franklin Lakes, NJ, USA) agar with nalidixic acid (32 µg mL^−1^; MP Biomedicals LLC, Solon, OH, USA) was used to enumerate nalidixic-resistant *E. coli* K12 Nal^R^. LB agar without antibiotic was used to enumerate *E. coli* O157:H7. mEnterococcus broth (Becton, Dickinson and Company, Franklin Lakes, NJ, USA) agar plates were used to enumerate *Enterococcus faecalis*. Xylose lysine deoxycholate agar (XLD agar; Hardy Diagnostics, Santa Maria, CA, USA) was used to enumerate Salmonella. Unless noted otherwise, experiments were independently repeated three times. To assess the potential for use in turbid liquids, *E. coli* K12-Nal^R^ (10^6^ cfu mL^−1^) (in 5 mL sterile water) was mixed with 5 g of loess soil [24] before or after iodine perfusion, followed by bacterial quantification.

For the bucket system the same strains and final concentrations were used. Ambient air was moved through the cartridge and diffuser by hand pumping for 2 min followed by a 5 min break and then for 2 additional min. After treatment, a 2 mL sample was used for bacterial enumeration. Total cfu counts were determined after each two-minute pumping session. The system was rested and after 24 h, a sample was collected before adding respective bacterial strains again at 10^6^ mL^−1^. After 5 min water samples were again collected for enumeration. The latter procedure was used to determine residual activity of iodine species after 24 h.

### 2.4. Biofilm Treatment

To assess the efficacy of the I_2_VP technology on antimicrobial resistant and biofilm forming bacteria, *Acinetobacter baumannii* (ATCC BAA-1605) and *Staphylococcus aureus* (ATCC BAA-1747) biofilms were grown separately for 24 h in 3 mL of LB broth. Cultures were incubated in 16 mL polystyrene tubes and grown without shaking at (37 °C). The LB broth was decanted and biofilms were rinsed by gently mixing with sterile water (4 mL). Rinse water was decanted, and the bottom 1 cm of each plastic tube was cut to yield an open-ended column. Control biofilm communities were harvested for enumeration by first inserting the 16 mL open-ended tube into a 50 mL polypropylene tube. Ten sterile glass beads and 3 mL of LB agar were added directly to the 16 mL tube and the entire apparatus was vortexed for 60 s to dislodge and disrupt any biofilm from the surface of the 16 mL tube. Cfu counts were conducted as described above. Immediately after rinsing, biofilm communities from the no-treatment tubes were vortexed and enumerated. Remaining treatments involved submersion of the biofilm tubes into 45 mL water within a 250 mL cylinder. Air with or without iodine vapor was passed through the system so that the biofilm communities were directly exposed to I_2_ vapor for 90 s. Vortexing with sterile beads and enumeration followed as described above.

### 2.5. Direct Infusion into Untreated Municipal Wastewater

To test applicability of the device as an alternative method of municipal water treatment, raw wastewater (sewer water) was collected from a municipal wastewater treatment plant (Pullman, WA, USA). Water was collected after removal of solid waste at the plant. A Bostitch trim Air 2.8CFM High Output compressor was used to move air through the iodine matrix followed by diffusion into 3-L wastewater samples (*n* = 3) for 2, 4, 8 and 16 min. The bucket was thoroughly cleaned with 70% ethanol between each treatment. After treatments serial dilutions were prepared and cfu mL^−1^ was estimated by using a 6X6 drop-plate method (see above).

### 2.6. Infusion into River and Pond Water

To assess efficacy of the device to clear naturally occurring bacteria from natural water bodies, we used pond and river water. We used the Bostitch trim Air 2.8 CFM High Output compressor with pumping for 2, 4, 8 and 16 min followed by enumeration by using both LB and MacConkey agar plates (Hardy Diagnostics, Santa Maria, CA, USA). MacConkey agar plates were used to selectively detect lactose and non-lactose fermenting Gram-negative bacteria. The same water samples were also treated using the hand pump. To challenge this system water was inoculated with three of the mentioned bacterial strains *E. coli K-12, Salmonella enterica* and *Enterococcus faecalis* as described above.

### 2.7. Infusion of Iodine Vapor into Dairy Lagoon Water

To assess the feasibility of using iodine to treat agricultural water, we collected water from the animal waste lagoon at Knott’s Dairy, Washington State University, Pullman, WA. Water was divided into two groups containing 20 mL aliquots. Water from the first group was sieved to remove bulk debris and then centrifuged at 3000x *g* for 15 min with the supernatant being subsequently treated as described above. The second group was not centrifuged. Experiments were conducted using the Bostitch trim Air 2.8 CFM High Output compressor with pumping for 4 and 8 min (air alone or with iodine vapor) followed by enumeration by using both LB and MacConkey agar plates (Hardy Diagnostics, Santa Maria, CA, USA). MacConkey agar plates were used to selectively detect lactose and non-lactose fermenting Gram-negative bacteria. This experiment was then repeated with 1 L of lagoon water to observe the effect of volume and continuous iodine exposure on bacteria recovery. Pumping was extended to 16 min.

### 2.8. Iodine Residue Testing

Water samples were collected, before pumping, at 2 and 4 min, and 24 h after infusion of iodine by either the compressor or hand pump in 25 mL glass tubes that were filled completely to exclude any air. These samples were then shipped by overnight courier to the Diagnostic Center for Population and Animal Health at Michigan State University for quantification of iodine species by using gas chromatography.

## 3. Results

### 3.1. Iodine Infusion is Effective Against Gram-Negative and -Positive Bacteria

**Figure 4:**
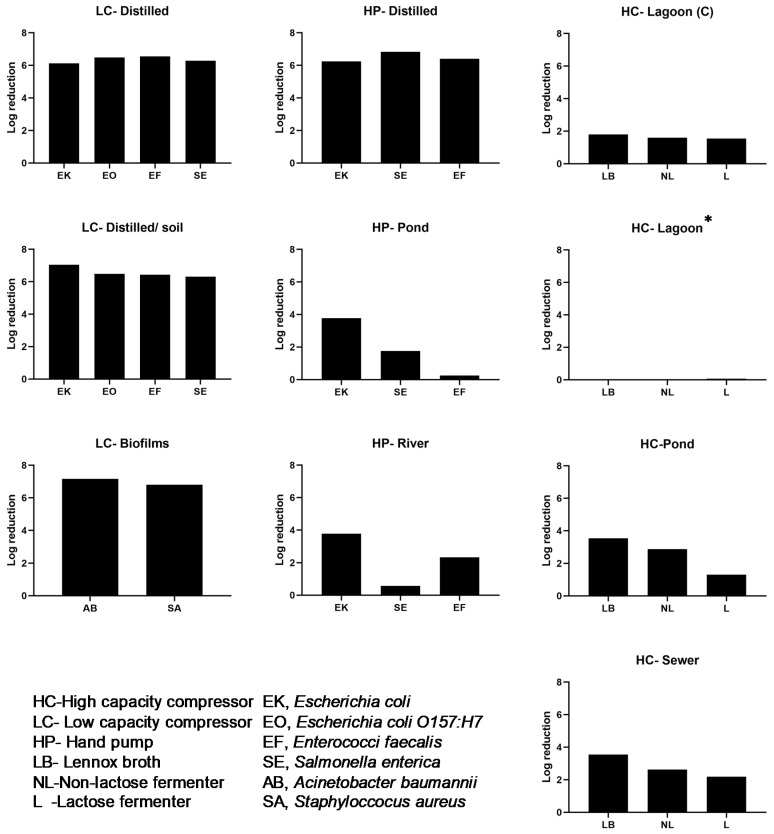
Log reduction following iodine treatment. **Left panel**: results after use of the low capacity compressor configuration to treat distilled water (LC-Distilled) inoculated with common water pathogens, distilled water plus loess soil (LC-Distilled/soil) and distilled water with biofilms (LC- Biofilms). A 6 log reduction of pathogens was observed using this system. **Middle panel**: results after use of hand pump configuration to treat distilled water (HP-Distilled), water from naturally occurring water bodies (HP-Pond) and (HP-River) inoculated with and common water pathogens. Organic content in pond and river water reduced efficacy of the device. **Right panel**: high capacity compressor configuration was used to treat water from naturally occurring water bodies (lagoon (HC-lagoon (centrifuged) and (HC-lagoon) pond (HC-pond) and sewer (HC-Sewer)) to simulate use of the technology in real settings with high particulate matter or organic matter. Even in the presence of increased iodine concentration organic matter still reduced efficacy of iodine. * cfu mL^−1^ used to determine log reduction was estimated by using a 6X6 drop-plate method with a limit of detection between 7–18 CFU/mL^−1^.

The low capacity compressor attached to the 250 mL cylinder was used for initial testing. We considered two modes of operation including “pre-infusion of iodine” before introduction of bacteria, or infusion after introduction of bacteria. Pre-infusing distilled water with iodine before adding approximately (10^6^ cfu mL^−1^) bacteria was sufficient to kill all four of the strains after only 90 s exposure (Table S1). However, since this had no practical applicability, in all experiments, unless stated, iodine treatment was done after addition of inoculum. When ambient air was infused without iodine, bacterial counts were within 0.2 log cfu mL^−1^ of the estimated inoculum value. When water with bacteria was infused directly with iodine, complete killing (6 log reduction) was observed with as little as 10 s infusion (Figure 4, left panel). The cfu mL^−1^ used to determine log reduction was estimated by using a 6X6 drop-plate method with a limit of detection between 7–18 CFU/mL^−1^ [32]. To test the effectiveness of iodine treatment in the presence of organic material, water was mixed with a sample of loess soil and bacterial inoculum was added before or after iodine infusion. Most of the *E. coli* K12-Nal^R^ was recovered after being mixed into the water-soil slurry that was pre-infused with iodine for 90 s (Table S1). Iodine treatment after addition of inoculum, however, completely removed the bacteria that were mixed with a starting inoculum of approximately 6 log cfu mL^−1^ (Table S1).

### 3.2. Iodine Infusion Eliminated Biofilm Communities

The low capacity configuration was also able to sterilize inoculum of biofilm forming multi-drug resistant *Acinetobacter baumannii* and methicillin resistant *Staphylococcus aureus*, important hospital pathogens by a 6-log reduction (Figure 4, left panel). To determine if infused iodine bubbles affects biofilm communities, *A. baumannii* and *S. aureus* biofilms were grown for 24 h as static cultures. Untreated *A. baumannii* biofilm yielded 7.16 log cfu mL^−1^ that was reduced to 5.32 log cfu mL^−1^ from air infusion alone (presumably due to shear force). Infusion with iodine vapor resulted in complete loss of *A. baumannii* after a brief (90 s) exposure (Table S1). Results for *S. aureus* biofilms were similar, with some loss of bacteria from air infusion alone (6.81 to 6.14 cfu mL^−1^), but complete loss of recoverable bacteria when infused with iodine vapor (Table 1).

For the hand-operated bucket system, water inoculated with *E. coli* K12-Nal^R^, *Salmonella*, or *Enterococcus* strains (Figure 4, middle panel) was exposed to air with and without iodine vapor treatment. For the volume treated (3 L) and inoculum present (app. 10^6^ cfu mL^−1^), infusion for 4 min was enough to completely sanitize the water of all bacteria that had been added (Table S2). Iodine-treated water remained sterile for at least 24 h. Subsequent addition of *Enterococcus faecalis* (8.58 log cfu mL^−1^) or *S. enterica* (9.87 cfu mL^−1^) to 24 h post-treated water resulted in only a limited reduction (approximately 2-log) in viable cells after 5 min (6.38 and 7.03 log cfu mL^−1^, respectively). Suggesting that residual iodine species were not high enough to maintain disinfection. For the bucket system, pond and river water were used to represent examples of undefined organic matter contamination. Given a volume of 3 L and up to 16 min hand pumping, bacterial counts were reduced 0.58 to 3.77 log cfu mL^−1^ from starting inoculum of approximately 6 log cfu mL^−1^ (Table S2). This suggests that organic matter limits the efficacy of iodine vapor.

### 3.3. Wastewater Treatment Produced Variable Results

After seeing reduced efficacy of iodine in low quality water, we decided to increase iodine concentration by using a high capacity compressor. Lagoon, pond and river water was used. We did not artificially inoculate the water but assessed naturally occurring bacteria. For this experiment we enumerated total bacteria on LB or MacConkey agar, with the latter being used to quantify lactose-fermenting or non-fermenting bacteria. Dairy lagoon wastewater was collected from a location where the water was manually agitated on a continuous basis resulting in a turbid sample with high organic load from sediment, fecal waste and other debris. Because lagoon water foamed, these experiments were conducted in a total volume of 20 mL (250 mL cylinder system) or 1 L (bucket with high-volume compressor). For the smaller volume of centrifuge lagoon water, total counts on LB did not change after an initial 1.8-log decrease, while counts using MacConkey agar did not decline 1.5 log until after 16 min continuous bubbling (Table S3). For the larger volume of uncentrifuged lagoon water, iodine infusion had no effect (Figure 4, left panel). This shows that in the presence of increased iodine concentration organic matter still reduced efficacy of iodine.

Municipal wastewater was collected at the inlet at the public works and 3-L samples were treated for each experiment. Municipal wastewater (sewer) had far less suspended solids (visual inspection) and had relatively low bacterial counts (≤3.5 log cfu mL^−1^) that were eliminated with as little as 4 min of iodine infusion (Table S3) (Figure 4, left panel). The pond water that we sampled had an initial bacterial count that was similar to municipal wastewater and was also eliminated with as little as 4 min of iodine infusion (Table S3).

### 3.4. Iodine Residues after Iodine Infusion

To test the iodine residue levels, water samples were collected before pumping 2 and 4 min, and 24 h thereafter for the hand pumped bucket system. The maximum concentration of iodine was detected after 4 min (0.183 ug mL^−1^), which decreased to 0.159 ug mL^−1^ after 24 h (Table 1). When a 3-L.

Volume of water was infused by using an electric compressor, residues peaked at 3.47 ug mL^−1^ after 16 min of iodine infusion. Samples collected from a 50 mL volume of water (using the 250 mL cylinder system) had the highest concentration of residues (16 min infusion, 12 ug mL^−1^ iodine). As anticipated, iodine residues were dependent on the volume of water being treated and the volume of air with iodine that was passed through the water

## 4. Discussion

This study describes the potential application of a newly patented technology (I_2_VP) that delivers iodine vapor for water disinfection. The technology was effective when sterilizing distilled water that was inoculated with enteric organisms (*Escherichia*, *Salmonella* and *Enterococcus*). This worked well when distilled water was pre-infused with iodine before adding bacteria, but also when water was infused. In drinking water, coliforms, *Escherichia coli* and enterococci have been used as the primary method of assessing contamination (representative strains were used in this study [32]. We show that the I2VP would be effective in sterilizing these coliforms by a 6-log reduction. The device was also effective against drug resistant pathogens tested i.e., methicillin-resistant *Staphylococcus* and multi-drug resistant *Acinetobacter* biofilm communities. This is also consistent with published reports of its potential use in controlling biofouling or for sanitation of interior surfaces of hoses [22].

The last comprehensive study of the disinfection efficacy of iodine for water treatment showed that concentrations in the range 5–10 ppm were effective against different types of microorganisms within 10 min at room temperature [6], another group showed that iodine tablets needed a contact time of 5–25 min depending on temperature [11] and up to 30 min in the presence of organic matter [33]. In this study, we were able to show efficacy of less than 90 s at room temperature with residues of 12 ppm with the electric power compressor and 4 min with residues less than 1 ppm following use of the hand pump. The electric powered compressor was superior in terms of time and effort required to deliver I_2_ vapor compared to the manual version. However, a 4 min pump time with the manual device was still less than half of the 10 min reportedly necessary to kill microorganism using aqueous iodine. Our findings suggest that the I_2_VP technology greatly reduces contact time necessary for water disinfection.

As has been also shown previously [33], application of this technology is limited when water contains a high level of suspended solids and organic materials such as manually agitated dairy waste lagoon water that we tested. The presence of natural organic substances is associated with iodine demand and reduced efficacy [7]. Even though this occurs, iodine shows appreciable lower reactivity when compared to chlorine, which is widely used for water disinfection [7]. Despite this limitation, we found the technology was effective against bacteria found naturally in municipal sewer water and in a sample of pond water provided that enough volume of I_2_ vapor is introduced into the water column. Depending on the situation, pre-filtration using a gravity-feed sand filter or using flocculation could be used as simple methods to reduce the concentration of suspended particles prior to I_2_ vapor treatment. Use of sequential UV radiation can also be considered as it has been shown to increase efficacy of chlorine in the presence of suspended solids [34]. Coupling iodine use with alternative disinfectants such as Peracetic acid (PAA) may also be explored [35].

We hypothesize that the technology works well because vaporized I_2_ is protected within the air bubbles long enough to be dispersed throughout a water column where it diffuses into the water and interacts with bacterial membranes. If correct, this is also consistent with reduced performance with increasing load of dissolved solids with which the iodine can interact. It also suggests that there will be optimal performance conditions that match the rate of infusion with water volume and concentration of interfering contaminants. Others have shown that iodine disinfection can be very sensitive to organic nitrogenous contaminants [10,11] and we surmise that the failures that we observed were due to the presence of organic compounds in the culture media. If correct, empirical testing will be needed to validate potential applications of this technology for different waste streams.

Hydrolysis and the subsequent equilibrium between elemental iodine and hypoiodous acid are pH-dependent, but the effect is not as pronounced as with chlorine [6], which allow iodine to be used across a wide range of pH. For our experiments the pH was neutral to mildly alkaline (pH 7–7.5), which is reportedly compatible with both elemental iodine and hypoiodous acid [7]. Elemental iodine is primarily effective against bacterial spores and protozoan cysts, whereas hypoiodous acid is known to be an effective bactericide and virucide [36]. We surmise that the I_2_VP technology can effectively deliver iodine to kill viruses and cysts, but this will need to be investigated further.

In addition, iodine is reportedly less reactive than chlorine and consequently chlorine is relatively less effective in the presence of organic material [37]. Because chlorine interacts with dissolved organic matter, it potentially forms harmful disinfection byproducts (e.g., trihalomethanes and haloacetic acids), most of which are regulated by the U.S. Environmental Protection Agency [37,38]. With respect to toxicity, recent research performed during a Navy Environmental Sustainability Development to Integration (NESDI) program compared the toxicity of aqueous chlorine and iodine by evaluating the relative effects on fertilization rates of the *Trepnuestes gratilla*, the Hawaiian collector sea urchin. Results were expressed as the no observable effect concentration (NOEC) and lowest observable effect concentration (LOEC). The NOEC for iodine and chlorine was 0.01 ppm and 0.002 ppm, respectively, while the LOEC for iodine and chlorine was 0.014 ppm and 0.003 ppm, respectively. These data are consistent with chlorine toxicity being an order-of-magnitude greater than that of iodine. Formation of DBPs from a number of iodine-based disinfectants was compared and it was found to be dependent on method of delivery method [39]. This report suggested that use of iodine tincture was associated with higher levels of iodoforms. This will require further investigation for the I_2_VP device. This suggests that perfecting iodine delivery will allow its use as a safer alternative to chlorine for water treatment.

Our results are consistent with scalability of the I_2_VP system, which is not surprising given that delivery and dispersion will be concentration and volume dependent. Larger diffusers, more air flow and longer exposures could be used to improve performance against larger volumes of water or when organic loads are higher. Nevertheless, under ideal conditions (i.e., with no visible contaminants) sanitation can be successful at a scale (3 L) that can be processed using a manually operated air pump for as little as 4 min. Given longer exposures and smaller volumes, the total residue concentration increases; hand-pumped bucket system (0.183 ug mL^−1^ for 3 L and 4 min), compressor pumped bucket system (3.47 ug mL^−1^ for 3 L; 16 min), 250 mL compressor cylinder (12 ug mL^−1^ for 250 mL, 16 min).

Iodine is an essential nutrient required for development and functioning of the thyroid gland, but excess iodine may lead to thyroid disease [40,41]. Toxicity to iodine has been linked to genetic or physiological predisposition (Rose et al., 2002) and to the use of iodine-based products as disinfectants [4] and medications [42]. Earlier work suggested that consumption of conventionally iodinated drinking-water did not cause adverse health effects in people [43]. More recent reports indicate that iodine toxicity is uncommon [44] and people appear to have a high tolerance to iodine given doses <2 mg day^−1^ [42]. The World Health Organization estimates that oral doses of 2000–3000 mg iodine (about 30–40 mg kg^−1^ of body weight) are lethal to people [45] while chronic ingestion of 2 mg of iodide per day (0.03 mg kg^−1^ of body weight per day) is considered excessive. Others have reported that daily doses of 50–80 mg (0.8–1.3 mg kg^−1^ of body weight per day) can be consumed without ill effect [46]. For the current study, if we assume that water consumption for an adult person is 4 L day^−1^ then water prepared by using the hand-pumped bucket system would deliver a daily dose of 0.73 mg of iodine that is well below suggested limits for chronic exposure. At the very least, the technology should be suitable for emergency water treatment in the absence of electricity assuming that the treated water has limited suspended solids. If higher air volume methods are used, real-time testing for iodine residues or secondary sequestration (e.g., activated charcoal) may be needed to guard against excessive iodine exposure.

The I_2_VP technology has also been tested elsewhere. A study funded by the US Navy demonstrated the potential for the patented I_2_ infusion system to reduce the rate of bio-fouling within Department of Defense shipboard heat exchangers [23,47]. For this project, periodic infusion of air containing elemental iodine vapor into the heat exchanger reduced the need for routine physical cleaning (which requires hazardous chemicals) while maintaining with acceptable operating parameters. Based on our findings, it is likely that this technology can be used for emergency and household drinking water treatment, sanitation of waste streams such as municipal sewage, and for managing biofilms that form within water lines that are used in food manufacturing and food animal agriculture.

## 5. Conclusions

Iodine infusion was effective against bacteria and biofilms even in water with moderate loads of suspended particles. It is unlikely to work well if treating waters containing high concentrations of dissolved solids (e.g., dairy lagoon water). The technology is relatively simple to implement and can be scalable for use as emergency water treatment and for reducing microbiological contamination of some waste streams such as municipal and hospital sewer lines. The technology is of low cost and we show that both an electric and hand operated version is effective in the absence of organic matter.

## Figures and Tables

**Table 1:** Iodine residues following hand pump and compressor delivered iodine vapor.

System Used	Condition	ug mL^−1 a^ (ppm)
Bucket system (3-L vol.)	No infusion	0
	2 min infusion	0.082
	4 min infusion	0.183
	24 h after 4 min infusion	0.159
Compressor (3-L vol.)	4 min infusion	1.42
	8 min infusion	2.19
	16 min infusion	3.47
	24 h after 16 min infusion	1.33
Compressor (50 mL vol.)	4 min infusion	8.46
	8 min infusion	9.52
	16 min infusion	12

^a^ Value is an average of three experimental replicates.

## Supplementary Materials

The following are available online at https://www.mdpi.com/1660-4601/17/10/3489/s1, Table S1: Log cfu ml^−1^ before and after treatment for 90 s with the 250-mL cylinder system, Table S2: Log cfu mL^−1^ before and after treatment at respective times with hand-pump bucket system (3 L test volume), Table S3: Log cfu mL^−1^ of naturally occurring bacteria before and after treatment of different water samples (3 L fluid volume, high air volume).
