# Supplementary material for: Deploying Elemental Iodine in a Vapor Form to Disinfect Water and to Clear Biofilms
Source: Int J Environ Res Public Health. 2020 May 16;17(10):3489. doi: 10.3390/ijerph17103489 (PMC7277347; doi:10.3390/ijerph17103489)
Supplement: Supplementary file 1 [file ijerph-17-03489-s001.pdf]

International Journal of
Environmental Research and Public Health

MDPI

Table S1. Log cfu ml^{-1} before and after treatment for 90 s with the 250-mL cylinder system.

Sample	Inoculum	Before Treatment	Iodine Pre-Treated Water [a]	Iodine Treatment after Addition of Inoculum [b]
Water	*Escherichia coli* K-12	6.13 (0.13) [c,d]	-	-[e]
	Escherichia coli O157:H7	6.49 (0.01)	-	-
	Enterococcus fecalis	6.56 (0.09)	-	-
	Salmonella enterica	6.28 (0.12)	-	-
Water & soil	*Escherichia coli* K-12	7.04 (0.20)	6.50 (0.01)	-
	Escherichia coli O157:H7	6.49 (0.01)	ND	-
	Enterococcus fecalis	6.43 (0.19)	ND	-
	Salmonella enterica	6.31 (0.04)	ND	-
Biofilms	*Acinetobacter baumannii*	7.16 (0.09)	ND	-
	Staphylococcus aureus	6.81 (0.23)	ND	-

[a] I_2 vapor was infused 90 s before addition of bacteria. Bacteria were enumerated 90 s later.
[b] Bacteria were added 10 s before I_2 vapor infusion
[c] Average of biological triplicates.
[d] Standard error of means (SEM)
[e]-Below detection limit
[f] ND = Not determined

Table S2. Log cfu mL^{-1} before and after treatment at respective times with hand-pump bucket system (3 L test volume).

Water Source	Inoculum	Before Treatment	2 min	4 min	8 min	≥24 h
Distilled	*Escherichia coli* K-12	6.25 (0.05) [a]	-[b]	-	-	ND [c]
	Salmonella enterica	6.83 (0.07)	3.76 (1.11)	-	-	ND
	Enterococcus fecalis	6.41 (0.06)	3.33 (1.71)	-	-	ND
River	*Escherichia coli* K-12	6.55 (0.26)	6.46 (0.22)	6.23 (0.07)	2.78 (1.83)	2.34 (1.42)
	Salmonella enterica	6.87 (0.05)	6.77 (0.10)	6.70 (0.15)	6.30 (0.23)	3.85 (0.11)
	Enterococcus fecalis	6.18 (0.03)	4.22 (2.11)	3.89 (1.95)	3.84 (1.92)	3.66 (1.83)
Pond	*Escherichia coli* K-12	6.55 (0.26)	6.46 (0.22)	6.23 (0.07)	2.78 (1.83)	2.34 [d] (1.42)
	Salmonella enterica	6.77 (0.16)	6.71 (0.20)	6.68 (0.34)	5.01 (1.07)	4.09 [d] (0.75)
	Enterococcus fecalis	6.32 (0.07)	6.37 (0.04)	6.27 (0.04)	6.07 (0.27)	5.74 [e] (0.42)

[a] Value is an average of biological triplicates; standard error of means (SEM)
[b]-Below detection limit
[c] ND = not determined.
[d] 48 h incubation.
[e] 72 h incubation.

Table S3. Log cfu mL^{-1} of naturally occurring bacteria before and after treatment of different water samples (3 L fluid volume, high air volume).

Water Sample	Time (min)	Lennox Broth Agar	MacConkey Agar	
			Non-Lactose	Lactose
Lagoon [a]	0	6.01 (0.07) [b]	4.3	4.99 (0.01)
	4	4.38 (0.04)	4.34 (0.10)	4.23 (0.08)
	8	4.26 (0.02)	4.18 (0.10)	4.07 (0.01)
	16	4.21 (0.03)	2.66 (0.36)	3.44 (0.06)

Lagoon [c]	0	5.42 (0.02)	5.18 (0.02)	2.91 (0.01)
	4	5.42 (0.02)	5.28 (0.05)	2.91 (0.03)
	8	5.44 (0.04)	5.27 (0.05)	2.91 (0.07)
	16	5.41 (0.01)	5.23 (0.09)	2.84 (0.06)
Sewer [d]	0	3.54 (0.02)	2.63 (0.16)	2.18 (0.06)
	4	- [e]	-	-
	8	-	-	-
	16	-	-	-
Pond [d]	0	3.54 (0.02)	2.88(0.06)	1.302 (0.66)
	4	-	-	-
	8	-	-	-
	16	-	-	-

[a] 20 ml of centrifuged dairy lagoon water was used.

[b] Value is an average of biological triplicates; standard error of the mean (SEM).

[c] 1 L of dairy lagoon water (not centrifuged) was used.

[d] 3 L of sample water was used

[e]-Below detection limit
